# Sugary food and beverage consumption and epithelial ovarian cancer risk: a population-based case–control study

**DOI:** 10.1186/1471-2407-13-94

**Published:** 2013-02-27

**Authors:** Melony G King, Sara H Olson, Lisa Paddock, Urmila Chandran, Kitaw Demissie, Shou-En Lu, Niyati Parekh, Lorna Rodriguez-Rodriguez, Elisa V Bandera

**Affiliations:** 1 The Cancer Institute of New Jersey, Robert Wood Johnson Medical School, 195 Little Albany St, NJ 08903, New Brunswick, USA; 2 School of Public Health, University of Medicine and Dentistry of New Jersey, Piscataway, NJ, USA; 3 Department of Epidemiology and Biostatistics, Memorial Sloan-Kettering, Cancer Center, New York, NY, USA; 4 New Jersey Department of Health, New Jersey State Cancer Registry, Trenton, NJ, USA; 5 Department of Nutrition, Food Studies and Public Health & Department of Population Health, Langone School of Medicine; New York University, New York, NY, USA

**Keywords:** Ovarian cancer, Diet, Sugar, Sugary foods, Sugary drinks, Added sugars, Caloric sweeteners, Case–control, Nutrition, Risk factors

## Abstract

**Background:**

Ovarian cancer is the deadliest gynecologic cancer in the US. The consumption of refined sugars has increased dramatically over the past few decades, accounting for almost 15% of total energy intake. Yet, there is limited evidence on how sugar consumption affects ovarian cancer risk.

**Methods:**

We evaluated ovarian cancer risk in relation to sugary foods and beverages, and total and added sugar intakes in a population-based case–control study. Cases were women with newly diagnosed epithelial ovarian cancer, older than 21 years, able to speak English or Spanish, and residents of six counties in New Jersey. Controls met same criteria as cases, but were ineligible if they had both ovaries removed. A total of 205 cases and 390 controls completed a phone interview, food frequency questionnaire, and self-recorded waist and hip measurements. Based on dietary data, we computed the number of servings of dessert foods, non-dessert foods, sugary drinks and total sugary foods and drinks for each participant. Total and added sugar intakes (grams/day) were also calculated. Multiple logistic regression models were used to estimate odds ratios and 95% confidence intervals for food and drink groups and total and added sugar intakes, while adjusting for major risk factors.

**Results:**

We did not find evidence of an association between consumption of sugary foods and beverages and risk, although there was a suggestion of increased risk associated with sugary drink intake (servings per 1,000 kcal; OR=1.63, 95% CI: 0.94-2.83).

**Conclusions:**

Overall, we found little indication that sugar intake played a major role on ovarian cancer development.

## Background

Ovarian cancer is the ninth most common cancer among women and ranks fifth in overall cancer deaths in women in the United States [1]. Ovarian carcinogenesis is multifactorial and genetic, environmental, and hormonal factors have been implicated [2]. Although the relationship between diet and ovarian cancer has been extensively evaluated, results are generally inconclusive [3,4]. Few studies [5-11] have examined the relationship between sugary foods and beverages and risk of ovarian cancer with inconclusive results. Furthermore, only one study investigated the effects of added sugars on ovarian cancer risk, finding an inverse association [12].

The World Cancer Research Fund/American Institute for Cancer Research (WCRF/AICR) Second Expert Report recommendations for cancer prevention include limiting consumption of refined sugars [13]. Nevertheless, the consumption of caloric sweeteners has increased rapidly in the United States over the past three decades [14]. Even with a recent drop in added sugar consumption by Americans older than 2 years, it still accounts for almost 15% of total energy intake [15]. This exceeds the 2010 Dietary Guidelines for Americans that recommend limiting calories from solid fats and added sugars to 5 to 15% of total energy intake [15,16]. To our knowledge this is the first study to evaluate ovarian cancer risk in relation to the consumption of sugary foods and beverages, total and added sugar intakes, as well as potential effect modification by insulin-related factors. It also evaluates the relevance of the WCRF/AICR’s recommendation to reduce sugar consumption in relation to ovarian cancer prevention. Understanding how the consumption of sugar affects ovarian cancer risk may further elucidate the role of diet in ovarian cancer etiology, as well as provide some strategies for prevention of this deadly disease.

## Methods

### Study population

The New Jersey Ovarian Cancer Study is a population-based case–control study and has been described elsewhere [17-19]. In brief, eligible women were older than 21 years, able to speak English and/or Spanish, and residents of six contiguous counties in New Jersey (Essex, Union, Morris, Middlesex, Bergen, and Hudson). Cases were newly diagnosed, histologically confirmed cases of invasive epithelial ovarian cancer, identified by rapid case ascertainment by the New Jersey State Cancer Registry, a SEER Registry. Population controls from the EDGE (Estrogens, Diet, Genetics, and Endometrial Cancer) Study served as controls for this study and are described elsewhere [20,21]. Briefly, controls were identified via random digit dialing (RDD) if under 65 years of age and Centers for Medicare and Medicaid Services (CMS) and area sampling if age 65+ years and 55+ years, respectively. Recruitment of cases and controls occurred between July 2001 and May 2008. Women who had a hysterectomy or those who had a bilateral oophorectomy were not eligible as controls in the NJ Ovarian Cancer Study. Informed consent was obtained from all participants. This study has been approved by the Institutional Review Boards of the New Jersey Department of Health and Senior Services, Memorial Sloan-Kettering Cancer Center and University of Medicine and Dentistry of New Jersey (UMDNJ) Robert Wood Johnson Medical School.

### Data collection

Same study procedures and materials were used for cases and controls. Informed consent was obtained before the phone interview. Cases and controls completed a phone interview during which a questionnaire was administered ascertaining demographic characteristics and major risk factors for the disease such as hormone use, family history of cancer, reproductive history, medical history, and lifestyle factors up to a year prior to diagnosis (or date of interview for controls). A food frequency questionnaire (FFQ), the Block 98.2 FFQ (110 food items), was self-administered and returned by mail, along with waist and hip measurements (a tape measure and instructions were provided), and a mouthwash sample for DNA extraction.

We initially identified 682 eligible cases, of whom some were excluded as they were either deceased (n=61) or physicians advised us not to contact them (n=9). Additional cases were excluded if they could not be reached or no longer met eligibility requirements, such as a communication barrier or medical conditions that precluded participation (n=119). In total, 233 of the remaining 493 cases (47%) and 467 controls (40%) completed the phone interview. Participants were excluded from the analysis if their menopausal status was unknown or if they were missing other major covariates. Those who were postmenopausal but did not know their age at menopause were included in the analysis. Of the remaining cases and controls, 205 cases (88%) and 398 controls (85%) completed both the interview and FFQ. Eight of these controls were excluded from these analyses because both of their ovaries had been removed. There were no significant differences in major characteristics between those who did and did not complete the food frequency questionnaire.

### Processing of dietary data

Participants’ responses were converted to number of servings per day based on their reported frequency and portion sizes for sugary foods and beverages. Frequency was measured as ‘never’, ‘a few times per year’, ‘once per month’, ‘2-3 times per month’, ‘once per week’, ‘2 time per week’, ‘3-4 times per week’, ‘5-6 times per week’, and ‘everyday’ for most food items. For a few foods, ‘never’ and ‘a few times per year’ were combined into one choice: ‘never or a few times per year’ and the choice of ‘2+ times per day’ was added. Portion size for food items was measured in teaspoons, tablespoons, ounces, pounds, cups, pieces, patties, bowls or slices. Portion size for beverages was measured as number of cups, glasses, cans or bottles consumed.

Serving sizes were based on the guidelines listed in Reference Amounts Customarily Consumed (RACC) Per Eating Occasion: General Food Supply by the Food and Drug Administration (FDA) [22]. This document provides the amount of food typically consumed per eating occasion, and is based on the 1977-1978 and 1987-1988 Nationwide Food Consumption Surveys. When making assumptions about participants’ portion sizes consumed, we used the FDA’s assigned RACC values as a guideline. For example, we assumed that one doughnut (RACC=55 grams) is equivalent to one serving. Therefore, participants who reported usually eating one doughnut per occasion were assigned as eating one serving for this food item.

Next, we computed the number of servings of dessert foods with added sugars, non-dessert foods with added sugars, sugary drinks and total sugary foods and drinks for each participant. Total and added sugar intakes (g/day) were calculated for each relevant food item by multiplying the frequency of intake by the total/added sugar content per 100 grams of food.

Total sugars are the sum of both natural and added sugars in the diet [14]. Natural sugars, like fructose or lactose, are found in whole fruit, vegetables, or milk products, which also have nutrients and phytochemicals beneficial to an individual’s health [23]. Added sugars are all caloric sweeteners that have been added to foods or drinks during processing, preparation, and also consumed separately or at the table. Foods and beverages with added sugars tend to be high in calories and lacking essential nutrients [23]. Examples of added sugars are sucrose (i.e. table sugar), high fructose corn syrup, honey, molasses, and syrups [14,15,23]. Sugary foods and drinks are foods that have been processed, prepared, or consumed with added sugars [23]. Total and added sugar content values were based on the USDA Database for Added Sugars Content of Selected Foods [24].

Calculation of percent of calories from sweets and desserts (% kcal from sweets) included the following FFQ items: regular and low-fat ice cream, ice milk or ice cream bars, doughnuts or Danish pastry, regular or low-fat cake, sweet rolls or coffee cake, regular and low-fat cookies, pumpkin pie or sweet potato pie, other pie or cobbler, chocolate candy or candy bars, candy (not chocolate), soft drinks or sweetened bottled drinks like Snapple (not diet), sugar or honey added to coffee/tea, breakfast bars, granola bars or power bars, sweetened cereals, and jelly, jam or syrup. Information about the respondent’s consumption of diet drinks or use of non-caloric sweeteners (within foods or added at the table) was not collected.

### Statistical analyses

Descriptive statistics were computed for total and added sugars and food and drink groups. For all analyses, statistical significance was considered a p-value less than 0.05. To describe our study population, the distribution of major characteristics for cases and controls was tabulated. Two sample t-tests were used to compare cases and controls across continuous variables and chi-square tests were used for categorical variables. Age-adjusted logistic regression models were used to calculate odds ratios (ORs) and 95% confidence intervals (CIs) to compare ovarian cancer risk across major risk factors (except for age).

ANCOVA was used to calculate age-adjusted means to compare mean intake between cases and controls for each food and drink group: dessert foods, non-dessert foods, sugary drinks, total sugary foods and drinks, as well as total and added sugar intakes. Based on the distribution in controls, tertiles for the food and drink groups and total and added sugars intake were created and frequencies calculated across the tertiles. Age-adjusted and multiple unconditional logistic regression models were used to estimate ORs and 95% CIs for the food and drink groups and total and added sugar intakes.

Covariates considered in multiple logistic regression models include age (continuous), years of education (≤12, 13-16, >16), race/ethnicity (White, Black, Other, Hispanic-any race), age at menarche (>13, 12-13, ≤11), menopausal status (pre- or postmenopausal) and age at menopause for postmenopausal women (<40, 41-54, ≥55, age at menopause unknown), parity (0-1, 2, ≥3), oral contraceptive (OC) use (ever vs. never), hormone replacement therapy (HRT) use (never, unopposed estrogen only, any combined HRT), BMI (weight in kg/height in m^2^; continuous), smoking status (never, past, current) and pack-years (continuous) for ever smokers, physical activity measured in continuous metabolic equivalents (METs), tubal ligation (yes vs. no), dietary intakes of fiber, total fat and saturated fat, and diabetes (yes vs. no). We adjusted for total energy intake using the multivariate nutrient density method [25]. Specifically, we computed density measures for servings of sugary foods and/or drinks per 1,000 kcal of intake, as well as grams of total or added sugars per 1,000 kcal of intake and included daily caloric intake as a continuous variable in the multivariable models. Tests for trend were conducted by assigning to each tertile the median value of servings of sugary foods and/or drinks per 1,000 kcal or total or added sugar intakes (g/1,000 kcal) among controls. In addition, tertiles for percent of calories from sweets and desserts (i.e. dessert foods group) per day were created based on the controls, and frequencies calculated across these tertiles. Odds ratios and 95% CIs were calculated to assess ovarian cancer risk across these tertiles.

Overweight or obesity is a strong determinant of insulin resistance and hyperinsulinemia [26-30]. Additionally, central obesity [31,32], which is related to insulin resistance [33], has been shown to significantly increase risk of ovarian cancer. We hypothesized that insulin-related risk factors might modify the relationship between sugar intake and cancer risk. Thus, we explored effect modification by factors capable of affecting the body’s response to insulin production such as BMI (normal weight: <25 kg/m^2^ vs. overweight or obese: ≥25 kg/m^2^), waist-to-hip ratio (WHR; ≤0.85 vs. >0.85), or physical activity (< median vs. ≥median for controls). Odds ratios and 95% CIs were calculated for ovarian cancer risk across tertiles for total sugary foods and drinks and total and added sugars, stratified by these factors. Because the number of women with diabetes was too small to conduct separate analyses on them, we also repeated analyses excluding women diagnosed with diabetes. The Wald test was used to calculate p-values.

## Results

Selected demographic characteristics and risk factors are presented in Table 1. In our study population, participants were mainly white and most had at least a college education. Compared to controls, cases were younger (64.6 vs. 57.0 years, respectively; *p*<0.01, data not shown), more likely to be either nulliparous or uniparous and premenopausal at the time of diagnosis. Having two or more children, combined HRT use and having a tubal ligation were associated with lower risk of developing ovarian cancer.

**Table 1:** Selected characteristics of women participating in the NJ ovarian cancer study

	**Cases (*n*=205)**		**Controls (*n*=390)**		**Age-adjusted**
	**n (%)**		**n (%)**		**OR (95% CI)**
**Education**					
High school or less	61	(29.8)	132	(33.9)	1.00 (Ref)
College	93	(45.4)	159	(40.8)	0.90 (0.59-1.38)
Graduate school	51	(24.9)	99	(25.4)	0.76 (0.47-1.24)
**Race/ethnicity**					
White	179	(87.3)	343	(88.4)	1.00 (Ref)
Black	9	(4.4)	17	(4.4)	1.02 (0.42-2.44)
Other	8	(3.9)	17	(4.4)	0.82 (0.33-1.99)
Hispanic (any race)	9	(4.4)	11	(2.8)	1.13 (0.44-2.92)
Parity*					
0 – 1	97	(47.3)	92	(23.6)	1.00 (Ref)
2	60	(29.3)	136	(34.9)	0.45 (0.29-0.69)
≥3	48	(23.4)	162	(41.5)	0.42 (0.26-0.66)
**Oral contraceptive use**					
Never	85	(41.5)	192	(49.2)	1.00 (Ref)
Ever	120	(58.5)	198	(50.8)	0.88 (0.61-1.28)
**Use of HRT**					
Never	159	(77.6)	284	(72.8)	1.00 (Ref)
Unopposed E only	22	(10.7)	34	(8.7)	1.56 (0.86-2.83)
Any combined HRT	24	(11.7)	72	(18.5)	0.63 (0.38-1.06)
**Age at menarche**					
>13	41	(20.1)	98	(25.2)	0.81 (0.51-1.28)
12-13	117	(57.4)	200	(51.4)	1.00 (Ref)
≤11	46	(22.6)	91	(23.4)	0.75 (0.48-1.17)
Menopause status*					
Premenopausal	71	(34.6)	49	(12.6)	
Postmenopausal	134	(65.4)	341	(87.4)	
Age at menopause					
<40	5	(2.4)	14	(3.6)	0.77 (0.26-2.31)
41-54	86	(42.0)	239	(61.3)	1.00 (Ref)
≥55	12	(5.9)	36	(9.3)	0.99 (0.48-2.02)
Unknown	31	(15.1)	52	(13.3)	1.52 (0.91-2.56)
**BMI**					
Normal (<25)	91	(44.4)	180	(46.5)	1.00 (Ref)
Overweight (25-29.9)	54	(26.3)	122	(31.5)	1.07 (0.69-1.65)
Obese (30-34.9)	36	(17.6)	59	(15.3)	1.39 (0.83-2.32)
Very obese (≥35)	24	(11.7)	26	(6.7)	1.54 (0.82-2.89)
**Smoking status**					
Never	108	(52.7)	203	(52.1)	1.00 (Ref)
Past	78	(38.1)	149	(38.2)	1.12 (0.76-1.64)
Current	19	(9.3)	38	(9.7)	0.87 (0.46-1.62)
**Tubal ligation**					
No	175	(85.4)	314	(80.5)	1.00 (Ref)
Yes	30	(14.6)	76	(19.5)	0.59 (0.36-0.94)
**First degree relative with ovarian cancer**					
No	195	(95.1)	376	(96.4)	1.00 (Ref)
Yes	10	(4.9)	14	(3.6)	1.32 (0.55-3.17)

OR: Odds Ratio, CI: Confidence Interval.

* *p*<0.01 for frequencies.

Table 2 shows age-adjusted means for the consumption of sugary foods and drinks, as well as, total and added sugars. Cases were more likely than controls to consume dessert foods, non-dessert foods and sugary drinks, although these differences were not significant. However, cases had significantly greater mean total sugar intake (64.7 vs. 60.2 grams/1,000 kcal, respectively), as well as, higher added sugar intake (29.5 vs. 26.3 g/1,000 kcal, respectively) compared to controls, although the latter did not reach statistical significance.

**Table 2:** Age-adjusted means for sources of dietary sugars among women in the NJ ovarian cancer study

**Sources of dietary sugars**	**Mean (SE)**		***p***
	**Cases (n=205)**	**Controls (n=390)**	
**Total sugary foods & drinks (servings/1000 kcal)**	5.12 (0.10)	4.83 (0.07)	0.39
**Dessert foods (servings/1000 kcal)**	0.87 (0.07)	0.83 (0.05)	0.64
Doughnuts, Danish pastry	0.04 (0.01)	0.04 (0.00)	0.85
Cakes, sweet rolls, coffee cake	0.03 (0.00)	0.03 (0.00)	0.40
Cookies	0.37 (0.04)	0.39 (0.03)	0.25
Ice cream	0.06 (0.01)	0.05 (0.00)	0.03
Pumpkin pie, sweet potato pie	0.01 (0.00)	0.01 (0.00)	0.72
Other pies or cobbler	0.02 (0.00)	0.02 (0.00)	0.11
Chocolate candy, candy bars	0.08 (0.01)	0.07 (0.01)	0.11
Other candy, not chocolate	0.28 (0.04)	0.24 (0.03)	0.81
**Non-dessert foods (servings/1000 kcal)**	3.94 (0.08)	3.80 (0.06)	0.39
Entrees	0.70 (0.03)	0.59 (0.02)	<0.001
Canned fruit, dried fruits	0.04 (0.01)	0.04 (0.00)	<0.01
Pancakes, waffles, French toast, Pop Tarts	0.06 (0.01)	0.07 (0.01)	0.89
Breakfast bars, granola bars, Power bars	0.03 (0.01)	0.03 (0.01)	0.08
Cooked cereals	0.07 (0.01)	0.11 (0.01)	<0.001
Cold cereals	0.15 (0.02)	0.17 (0.01)	0.36
Yogurt/Frozen Yogurt	0.08 (0.01)	0.08 (0.01)	0.72
Biscuits or muffins	0.79 (0.04)	0.80 (0.03)	<0.01
Jelly, jam, or syrup	0.15 (0.01)	0.15 (0.02)	<0.01
Other condiments	1.61 (0.06)	1.53 (0.05)	<0.01
**Sugary drinks (servings/1000 kcal)**	0.30 (0.03)	0.24 (0.02)	0.17
Drinks with added vitamin C	0.01 (0.00)	0.01 (0.00)	0.35
Drinks with some fruit juices	0.01 (0.00)	0.01 (0.00)	0.97
Regular soft drinks or bottled drinks	0.12 (0.02)	0.09 (0.01)	0.18
**Total sugars (g/1000 kcal)**	64.66 (1.72)	60.15 (1.22)	<0.01
**Added sugars (g/1000 kcal)**	29.46 (1.12)	26.25 (0.80)	0.07

SE: Standard Error.

Multivariable analyses revealed an increased ovarian cancer risk associated with higher consumption of total sugary foods and drinks and sugary non-dessert foods after adjusting for age and energy intake (Table 3). However, these associations did not remain significant after further adjustment for additional risk factors. There was a suggestion of a 63% increase in risk associated with each additional serving of sugary drinks per 1,000 kcal after adjusting for all risk factors, but the confidence interval included the null value (OR=1.63, 95% CI: 0.94-2.83). Further adjustment for diabetes, fiber, total fat, or saturated fat intakes did not significantly change results (data not shown). We also evaluated the impact of total carbohydrate, glycemic index and glycemic load. While ORs were above one, confidence intervals included one. Adjusted ORs (95% CI) for high vs. low quartiles were 1.18 (0.68-2.03) for total carbohydrate, 1.23 (0.71-2.14) for glycemic index, and 1.59 (0.76-3.30) for glycemic load (data not shown).

**Table 3:** Sources of dietary sugars and ovarian cancer risk in the NJ ovarian cancer study

**Sources of dietary sugars**	**Cases (n=205)**	**Controls (n=390)**	**OR1**	**95% CI**	**OR2**	**95% CI**
**Total sugary foods & drinks (servings/1000 kcal)**						
*Continuous (per serving)*			*1.15*	*(1.02-1.31)*	*1.05*	*(0.90-1.22)*
<4.14	50 (24.4)	130 (33.3)	1.00		1.00	
4.14-5.37	74 (36.1)	130 (33.3)	1.45	(0.91-2.29)	1.25	(0.73-2.16)
>5.37	81 (39.5)	130 (33.3)	1.74	(1.10-2.74)	1.25	(0.73-2.17)
*p* trend				0.02		0.46
**Dessert foods (servings/1000 kcal)**						
*Continuous (per serving)*		*1.05*	*(0.87-1.27)*	*0.94*	*(0.75-1.17)*	
<0.35	68 (33.2)	132 (33.9)	1.00		1.00	
0.35-0.80	66 (32.2)	128 (32.8)	0.99	(0.64-1.54)	0.92	(0.55-1.56)
>0.80	71 (34.6)	130 (33.3)	1.24	(0.79-1.94)	1.04	(0.61-1.76)
*p* trend				0.29		0.81
**Non-dessert foods (servings/1000 kcal)**						
*Continuous (per serving)*		*1.10*	*(0.95-1.29)*	*1.02*	*(0.85-1.23)*	
<3.21	52 (25.4)	132 (33.9)	1.00		1.00	
3.21-4.20	73 (35.6)	128 (32.8)	1.47	(0.93-2.31)	1.30	(0.76-2.22)
>4.20	80 (39.0)	130 (33.3)	1.58	(1.01-2.48)	1.31	(0.77-2.24)
*p* trend				0.05		0.35
**Sugary drinks (servings/1000 kcal)**						
*Continuous (per serving)*		*1.53*	*(0.96-2.44)*	*1.63*	*(0.94-2.83)*	
<0.03	62 (30.2)	130 (33.3)	1.00		1.00	
0.03-0.21	64 (31.2)	129 (33.1)	0.91	(0.59-1.44)	0.83	(0.48-1.41)
>0.21	79 (38.5)	131 (33.6)	1.17	(0.76-1.82)	1.09	(0.65-1.84)
*p* trend				0.30		0.47
**Total sugars (g/1000 kcal)**						
*Continuous (per 5g)*			*1.04*	*(1.00-1.08)*	*1.03*	*(0.99-1.08)*
<49.33	68 (33.2)	129 (33.1)	1.00		1.00	
49.33-69.61	70 (34.2)	130 (33.3)	1.19	(0.77-1.84)	1.32	(0.78-2.25)
>69.61	67 (32.7)	131 (33.6)	1.31	(0.84-2.04)	1.13	(0.66-1.94)
*p* trend				0.24		0.69
**Added sugars (g/1000 kcal)**						
*Continuous (per 5g)*			*1.07*	*(1.01-1.13)*	*1.04*	*(0.97-1.11)*
<18.63	61 (31.2)	129 (33.1)	1.00		1.00	
18.63-29.59	65 (33.2)	131 (33.6)	1.01	(0.64-1.59)	1.03	(0.59-1.77)
>29.59	79 (35.6)	130 (33.3)	1.35	(0.87-2.09)	1.05	(0.61-1.79)
*p* trend				0.16		0.87
**% Kcal from sweets**						
<8.10	58 (28.3)	127 (32.6)	1.00		1.00	
8.10-15.10	63 (30.7)	127 (32.6)	1.11	(0.70-1.76)	0.84	(0.49-1.46)
>15.10	84 (41.0)	136 (34.9)	1.40	(0.89-2.19)	1.10	(0.63-1.92)
*p* trend				0.13		0.57

OR: Odds Ratio, CI: Confidence Interval.

OR1: adjusted for age (continuous), daily caloric intake (continuous).

OR2: additionally adjusted for education (high school or less, college, graduate school), race (White, Black, Other, Hispanic), age at menarche (continuous), menopausal status (premenopausal, postmenopausal) and age at menopause for postmenopausal women (<40, 42-54, ≥ 55, unknown), parity (0-1, 2, 3-4), oral contraceptive use (ever, never), HRT use (never, unopposed estrogen only, any combined HRT), tubal ligation (no, yes), BMI (continuous), smoking status (never, past, current) and pack-years for ever smokers (continuous), and physical activity (METs for reported average hours per week of moderate or strenuous recreational activities).

Stratified analyses by BMI, WHR, physical activity, oral contraceptive use and menopausal status were based on small numbers and did not provide clear evidence of effect modification (data not shown). We repeated analyses excluding HRT users and those with diabetes and results were similar (data not shown).

## Discussion

Our study provided little support for a relationship between ovarian cancer risk and intake of sugary foods and beverages or total and added sugars. There was a suggestion of a moderately increased cancer risk associated with each additional serving of sugary drinks per 1,000 kcal, however, the confidence interval included the null value.

Relatively few studies have previously evaluated the role of intake of sugary food and beverages and total added sugars on ovarian cancer risk (Tables 4 and 5). To our knowledge, sugary beverage intake and ovarian cancer risk has only been evaluated in a few population-based [6,11] and hospital-based [10] case–control studies. Among them, only the study by Kuper et al. conducted in Massachusetts and New Hampshire [11] reported an association, with women who consumed the highest level of caffeinated cola beverages having elevated risk of ovarian cancer.

**Table 4:** Characteristics of prospective cohort studies evaluating sugar consumption and ovarian cancer risk

**Reference**	**Location**	**Cases/cohort size (n)**	**Dietary assessment**	**Time frame of dietary assessment**	**Sugar variables**	**Covariates**	**Effect modifiers**	**Results**
Kushi et al., 1999 [7]	Iowa (United States)	139/29,083	FFQ (126 items), 24-hour dietary recall among a subset	Current intake at baseline	“Breads, cereals, starches”, sweets	age, energy intake, # of live births, age at menopause, family history of ovarian cancer in a 1st-degree relative, hysterectomy/unilateral oophorectomy status, WHR, physical activity, pack-years smoked, educational	None	*- association:* breads, cereals, starches *+ association:* sweets
Silvera et al., 2007 [34]	Canada	264/48776	FFQ (86 items)	Current intake at baseline	Total sugar	age, BMI, alcohol intake, HRT use, OC use, parity, age at menarche, menopausal status, energy intake, physical activity, fiber intake, study center, treatment allocation	Menopausal status, smoking history, age at menarche, HRT use, alcohol intake, parity	*No association:* total sugar *+association:* strong, suggested association with sugar among postmenopausal women No effect modification by smoking history, age at menarche, HRT use, alcohol intake, parity
Tasevska et al., 2012 [12]	8 states in USA (CA, FL, LA, NJ, NC, MI, GA, PA)	457/179,990	FFQ, DHQ (124 items)	1 year prior to index date	Total sugars, added sugar, sucrose, total fructose, added sucrose, added fructose	age, BMI, family history of cancer, marital status, smoking status and pack-years smoked, race, education, physical activity, energy intake, alcohol intake	HRT	*- association:* total sugars, added sugars, total fructose, sucrose, added sucrose, added fructose; no modification by HRT

Abbreviations: *FFQ*- food frequency questionnaire, *DHQ*- diet history questionnaire, *BMI*- body mass index, *HRT*- hormone replacement therapy, *ERT*- unopposed estrogen replacement therapy, *OC*- oral contraceptives, *WHR*- waist-to-hip ratio, “+ association” - positive association, “- association”- negative association.

**Table 5:** Characteristics of case–control studies evaluating sugar consumption and ovarian cancer risk

**Reference**	**Location**	**Cases/controls (n)**	**Dietary assessment**	**Time frame of dietary assessment**	**Sugar variables**	**Covariates**	**Effect modifiers**	**Results**
***Case–control studies: population-based***								
Kuper et al, 2000 [11]	MA, NH (United States)	549/516	FFQ plus open ended section for unlisted foods	1 year prior to index date	Caffeinated cola	Age, study center	Menopausal status, tumor histologic type	*+ association:* highest level of consumption of caffeinated cola No evidence of effect modification
McCann et al., 2003 [8]	NY (United States)	124/696	Interviewer-administered diet questionnaire (172 items)	12 month period 2yr before interview	Snacks	age, education, total months menstruating, difficulty becoming pregnant, OC use, menopausal status, energy intake	None	*No association:* Snacks
Pan et al., 2004 [9]	Canada	442/2,135	FFQ (69 items)	2 years prior to index date	Baked desserts	age, province of residence, education, alcohol consumption, pack-years smoked, BMI, total kcal, physical activity, # of live births, menstruation years, menopause status	None	*No association:* baked desserts
Kolahdooz et al, 2009 [6]	Australia	717/806	FFQ (123 items)	1 year prior to index date	“Meat and fat”^1^ category: High-energy drinks and sweetened food and sugar	age, age squared, OC use, parity, education, energy intake	Tumor stage	*No association*: high-energy drinks and sweetened food and sugar did not explain the relationship between “meat and fat” and ovarian cancer
Chandran et al., 2011 [17]	NJ (United States)	205/390	FFQ (110 items)	6 months prior to index date	SoFAAS: total calories from solid fat, alcoholic beverages, and added sugar	Age, education, race, age at menarche, menopausal status, parity, OC use, HRT use, tubal ligation, BMI, energy intake, physical activity, smoking status, pack-years smoked	None	*No association*: SoFAAS
Nagle et al., 2011 [35]	Australia	1,366/1,414	FFQ (136 items)	1 year or if diet changed in last 6-12 mo, their usual diet	Total sugar	age, OC use, education, parity, BMI, menopausal status, energy intake	BMI, HRT use, menopausal status	*No association:* total sugar + *association:* total sugars among overweight/obese women. No effect modification by HRT use and menopausal status
***Case–control studies: hospital-based***								
Tzonou et al., 1993 [36]	Greece	189/200	FFQ (110 items)	1 year prior to index date	Sucrose	Age, education, parity, age at first birth, menopausal status, energy intake	None	*No association:* sucrose
Bosetti et al., 2001 [5]^2^	Italy	1,031/2,411	FFQ (78 items, plus range of courses and dishes)	2 year prior to index date	Desserts, Sugar	age, study center, year of interview, education, parity, OC use, energy intake	None	*+ association:* sugar, *Borderline + association:* desserts
Bidoli et al., 2002 [37]^2^	Italy	1,031/2,411	FFQ (78 items, plus range of courses and dishes)	2 year prior to index date	Sugar	age, study center, year of interview, education, parity, OC use, energy intake	Parity, menopausal status, energy intake, age, education, OC use	*No association:* sugar No evidence of effect modification
Salazar-Martinez et al, 2002 [10]	Mexico	84/629	FFQ (116 items)	1 year prior to index date	Sucrose, fructose, glucose, maltose, “bread and cereal”, “sweets and desserts”, “soda, coffee, and tea”, tortilla	age, energy intake, # of live births, recent changes in weight, physical activity, diabetes	None	*No association:* sucrose, fructose, glucose, maltose, bread and cereal, sweets and desserts, soda, coffee and tea, tortilla

Abbreviations: *BMI*- body mass index, *DHQ*- diet history questionnaire, *ERT*- unopposed estrogen replacement therapy, *FFQ*- food frequency questionnaire, *HRT*- hormone replacement therapy, *OC*- oral contraceptives, *WHR*- waist-to-hip ratio, “+ association” - positive association, “- association”- negative association ^1^ “Meat and fat” category included processed and red meat, poultry, liver, high-energy drinks (Cola drinks, other soft drinks, and cordials) and sweetened foods (cake, tart or pie, pastry, pavlova (meringue dessert), cheesecake, sweet roll, bun, plain sweet biscuits, fancy biscuits (e.g. chocolate coated), chocolate, lollies (candies), jam, peanut butter, and sugar) ^2^ Bidoli (2002) and Bosetti (2001) were from the same study.

Only a few studies have reported on the impact of various sugary foods on ovarian cancer risk, with inconclusive results. Similar to our results, Pan et al. [9] using data from the Canadian National Enhanced Cancer Surveillance System (NECSS), a population-based case–control study in pre- and postmenopausal women, did not find an association with baked desserts after adjusting for multiple factors including BMI, total caloric intake, and recreational physical activity [9]. Salazar-Martinez et al. [10] also did not find an association with soda, coffee and tea combined (OR=0.96; 95% CI: 0.40-2.29) in their hospital-based case–control study in Mexico. It is worth noting, while the authors did adjust for total energy intake, recent changes in weight, physical activity (METs), and diabetes, they did not adjust for smoking status or pack-years, BMI or WHR. Similarly, two hospital-based case–control studies [5,10] that have evaluated sugary food intake and risk of ovarian cancer reported non-statistically significant increases in risk associated with dessert consumption. In contrast, Kushi et al. [7] found a strong adverse association between sweets and ovarian cancer risk in the Iowa Women’s Health Study, a prospective study of almost 30,000 postmenopausal women among whom 139 cases were identified during the follow-up period (ORs from lowest to highest category: 1.00, 2.32, 2.49, and 1.61; *p*_trend_=0.17].

Overall, studies have produced inconsistent findings on the relationship between dietary sugars (i.e. total sugars, added sugar sucrose or fructose) and ovarian cancer risk. Only two prospective studies [12,34] have evaluated the relationship between sugar intake and ovarian cancer with conflicting results. Interestingly, using data from the NIH-AARP Diet and Health Study, Tasevska et al. [12] found the risk of developing ovarian cancer to be significantly inversely associated with total sugars, total fructose, and sucrose [Hazard Ratio (HR) (95% CI) for Quartile 5 vs. Quartile 1, respectively: 0.70 (0.51-0.97); 0.68(0.49-0.95); and 0.65(0.47-0.89)]. Unlike our study, 97% of their 457 ovarian cancer cases were postmenopausal and the authors state that their results could be confounded by unknown factors. On the other hand, Silvera et al. [34] did not detect a relationship between total sugar intake and ovarian cancer risk among premenopausal women in a prospective cohort in Canada. However, they did report increased risk with total sugar intake (g/day) among postmenopausal women (HRs from lowest to highest category: 1.00, 1.67, 2.35, and 1.79; *p*_trend_=0.08). They also found no heterogeneity of effects among pre- or postmenopausal women by smoking status, parity, age at menarche, HRT use, or alcohol intake [34]. Finally, among the studies [5,10,12,17,34-41] that have evaluated sugar intake, only one study [12] independently evaluated the effects of added sugar on risk of developing ovarian cancer. Tasevska et al. [12] detected significant protection against ovarian cancer among women in the highest quintile of added sugars intake, after adjusting for multiple factors [HR=0.72, 95% CI: (0.51-1.00); *p*_trend_=0.02].

We also considered a potential effect modification by physical activity, central adiposity, and general obesity, and did not observe any significant heterogeneity of effects estimates. Abdominal obesity [31] and high WHR [32], both markers of insulin resistance [33], have been shown to significantly increase ovarian cancer risk. Furthermore, insulin encourages ovarian production of androgens (direct precursors of estrogen synthesis) [42-45] and controls metabolism and transport of androgens in peripheral tissue [45]. This results in lower levels of insulin-like growth factor-binding protein and consequently increases insulin-like growth factor-1, promoting ovarian carcinogenesis [32,34]. Insulin-related factors, like WHR, might also modify the relationship between sugar intake and cancer risk. Nagle and colleagues [35] found sugar intake to have a beneficial effect on ovarian cancer risk among normal weight women and an adverse effect among overweight and obese women. They hypothesized that among heavier women, insulin resistance would exaggerate the harmful metabolic responses with carbohydrate consumption. Thus, a high-sugar diet could possibly have a more deleterious effect on ovarian cancer risk among women who are obese [35]. However, we did not find consistent evidence that sugar consumption and ovarian cancer risk was negatively impacted by central adiposity or excess weight. The study of Nagle and colleagues [35] and our study are the only studies to evaluate possible effect modification by BMI and therefore additional studies are needed. However, our study had limited statistical power for these stratified analyses and results should only be viewed as preliminary.

Recent studies have reported significant differences across histologic subtypes in the associations of epithelial ovarian cancer with reproductive and non-reproductive risk factors, perhaps due to variations in etiology, morphology, and genetic expression of ovarian tumors [46,47]. Using data from the Nurses’ Health Study and Nurses’ Health Study II, Gates et al. observed that determinants such as age, duration of estrogen use, BMI, duration of breastfeeding, age at menopause, and smoking significantly differed by histologic subtype [46]. It is possible that our inability to detect a relationship between sugar intake, ovarian cancer, and insulin modifiers may be a result of variations in risk across subtypes, which we were not able to evaluate due to limited statistical power. To our knowledge, no other studies have reported on sugar intake and ovarian cancer risk by histological subtypes.

Some limitations of our study must be noted. First, portion sizes were based on national food surveys performed over twenty years ago. Using nationally representative data collected between 1977 and 1996, Nielsen and Popkin [48] observed notable increases in US portion sizes for several food items including desserts, soft drinks, and fruit drinks. Thus, it is possible that we underestimated sugar intake in both cases and controls, resulting in non-differential exposure misclassification and underestimation of the magnitude of the association. This is unlikely to have had a major impact in estimates, however, as most of the variance in intake is due to frequency and not portion size [25]. Additionally, recall and selection biases are a particular concern in case–control studies. Unlike our study, two prospective cohort studies reported adverse associations between sugar intake and ovarian cancer risk among postmenopausal women [7,34]. It is conceivable that in our study, cases tended to under-report their sugar intake. We also assessed whether selection bias might have occurred by comparing characteristics of our ovarian cancer cases with all women diagnosed with epithelial ovarian cancer in the same NJ counties [49]. Our study participants were younger than the general population of cases (median age at diagnosis: 56 years vs. 61 years, respectively). On the other hand, our cases were similar with respect to race and ethnic distribution, as well as, histology, stage, and grade of cancer. Similar to many other epidemiologic studies [50], our study suffered from low response rates (47% and 40% for cases and controls, respectively). One concern is that participation may be related to subjects’ lifestyle habits, particularly for controls. For example, those who chose to participate in our study may make healthier choices and be more enthusiastic about participating in a health study than those who refused. However, this issue is not unique to our study, but a reality in medical research. Unfortunately, we were unable to compare controls with women who did not participate as we did not collect information on those who could not be reached or declined to participate in our study. However, we were reassured that major selection bias may not have affected our study as the distribution of major risk factors is comparable to those reported in the literature.

## Conclusions

To our knowledge this is the first study to evaluate ovarian cancer risk in relation to total and individual consumption of sugary foods and beverages, total and added sugar intake, as well as a potential effect modification by several insulin-related risk factors. Although in our study there was a suggestion of a moderately increased cancer risk associated with sugary beverage consumption, overall, we did not detect significant relationships with any of the sugar variables evaluated. The overall evidence for sugary foods and drinks and added sugars remains inconclusive. These apparent gaps in the literature emphasize the need for future research, preferably large prospective studies, to evaluate the role of added sugars in the etiology of ovarian cancer, while taking into consideration various factors capable of influencing the body’s insulin response such as anthropometric measures and physical activity.

## Abbreviations

WCRF: World Cancer Research Fund International;AICR: American Institute for Cancer Research;SE: Standard error;OR: Odds ratio;CI: Confidence interval;FFQ: Food frequency questionnaire;BMI: Body mass index;WHR: Waist-to-hip ratio;HRT: Hormone replacement therapy;OC: Oral contraceptives;METs: Metabolic equivalents

## Competing interests

The authors declare that they have no competing interests.

## Authors’ contributions

MGK wrote the first draft of the manuscript. EVB conceptualized the study design and supervised the implementation of the study. MGK, EVB, and UC performed all the data analyses. SHO recruited members of the control group in conjunction with the EDGE Study. Additional expertise was provided by KD and SHO (cancer epidemiology), S-EL (biostatistics), NP (nutrition/nutritional epidemiology), and LRR (gynecologic oncology). All authors provided substantive comments and editorial review and approved the final version of the manuscript.

## Pre-publication history

The pre-publication history for this paper can be accessed here:

http://www.biomedcentral.com/1471-2407/13/94/prepub
